# Identification of Novel Inhibitors of a Plant Group A Protein Phosphatase Type 2C Using a Combined *In Silico* and Biochemical Approach

**DOI:** 10.3389/fpls.2020.526460

**Published:** 2020-09-16

**Authors:** Maciej Janicki, Małgorzata Marczak, Agata Cieśla, Agnieszka Ludwików

**Affiliations:** Laboratory of Biotechnology, Faculty of Biology, Institute of Molecular Biology and Biotechnology, Adam Mickiewicz University in Poznan, Poznan, Poland

**Keywords:** protein phosphatase 2C group A, structure-based virtual screening, PP2C inhibitor, protein-ligand complexes

## Abstract

Type 2C protein phosphatases (PP2Cs) of group A play a significant role in the regulation of various processes in plants including growth, development, ion transport, and stress acclimation. In this study, we selected potential PP2C group A inhibitors using a structure-based virtual screening method followed by biochemical and *in vitro* validation. Over twenty million chemical compounds from the ZINC database were used for docking studies. The precision of the calculations was increased by an induced-fit docking protocol and the molecular mechanics/generalized Born surface area (MM/GBSA) method, which yielded approximate values for the binding energy of the protein-ligand complex. After clustering and ranking their activity, the top-ranking compounds were tested against PP2C group A members *in vitro* and their *in vivo* activity was also explored. Phosphatase activity assays identified two compounds with significant inhibitory activity against ABI1 protein ranging from around 57 to 91% at a concentration of 100 μM. Importantly, this *in vitro* activity correlated well with *in vivo* inhibition of seed germination, as expected for PP2C inhibitors. The results should promote the design of novel inhibitors with improved potency against ABI1-like and other PP2Cs that might be used in agriculture for the protection of crops against stress.

## Introduction

Reversible protein phosphorylation is a key protein modification involved in the regulation of numerous plant cellular processes (Friso and Van Wijk, 2015; Gerotto et al., 2019). In Arabidopsis, 76 members of the protein phosphatase type 2C (PP2C) family have been identified, which can be divided into 10 groups according to sequence similarity and subdomain composition. Ongoing research has demonstrated that Arabidopsis group A PP2Cs (ABI1, ABI2, HAB1, HAB2, HAI1, HAI2, HAI3, PP2CA/AHG3, and AHG1) are important regulatory components of the ABA signaling pathway (Schweighofer et al., 2004; Miyazono et al., 2009; Nishimura et al., 2009; Fuchs et al., 2013). For example, ABI1-like PP2Cs play a significant role in the regulation of plant growth, development, ion transport, and stress acclimation (Himmelbach et al., 2002; Yoshida et al., 2006; Saez et al., 2008). Importantly, genetic analysis demonstrates that ABI1, HAB1, ABI2, and HAB2 are key regulators of drought tolerance (Saez et al., 2006; Bhaskara et al., 2012), and therefore the respective signaling pathways are good candidates for genetic engineering to improve crop tolerance to drought.

Group A PP2Cs, together with SnRK2s and the PYR/PYL/RCAR family of START proteins known as ABA receptors, define the core ABA signal transduction pathway (Miyazono et al., 2009; Cutler et al., 2010; Nishimura et al., 2010; Umezawa et al., 2010). In recent years, notable progress in ABA receptor research has been reported (Melcher et al., 2009; Miyazono et al., 2009; Miyakawa et al., 2013). In Arabidopsis, when ABA is absent, PP2Cs block ABA signaling *via* PYR/PYL/RCAR ABA receptors by inactivation of SnRK2s (Miyazono et al., 2009). In contrast, when ABA is present, PYR/PYL/RCAR ABA receptors interact with and inhibit group A PP2Cs, releasing SnRK2s from PP2C-dependent regulation. Under such conditions, SnRK2s are able to phosphorylate downstream targets. Crystallographic and biochemical studies of an ABA-PYL1-ABI1 complex revealed a gate-latch-lock mechanism of PYL-dependent inhibition of the ABI1 PP2Cs. The ABI1 structure in the complex shows a folding pattern typical of PP2C family proteins. ABI1 is composed of a central β-sandwich surrounded by five α-helices and a small domain combining β-sheet and helix α3. In the ABA-PYL1-ABI1 complex, ABI1 interacts with the receptor *via* a region around the active site and an additional small domain. A characteristic feature of the interaction between ABI1 and PYL1 is that the ABI1 active site is covered by the β3-β4 loop of PYL1. Additionally, the small domain of ABI1 projects from the surface and interacts with the hydrophobic pocket of the ABA-PYL1 complex. When ABA binds PYL1, ABA-induced conformational changes in two β-loops (which serve as a gate and latch) close the gate and create a surface that enables the receptor to dock into the ABI1 active site. As a consequence, a conserved tryptophan residue (Trp300) in ABI1 inserts between the gate and latch to lock the PYL1 receptor (Melcher et al., 2009; Miyazono et al., 2009; Miyakawa et al., 2013). PYL1 inhibits ABI1 enzyme activity by covering the active site cleft; thereby preventing access of the substrates phosphoserine and phosphothreonine. Ser112 of PYL1 contacts Gly180 and Glu142 of ABI1 *via* water-mediated hydrogen bonds. Other residues from the β3-β4 loop of PYL1 are also located near the ABI1 active site. The interaction between ABI1 and PYL1 results in conformational changes that decrease the area of the ABI1 active site (Miyazono et al., 2009). In addition, substitution of Gly180 with Asp in ABI1 seems to reduce intermolecular interaction at this site due to the bulky acidic side chain of Asp; this significantly decreases, although does not abolish, the phosphatase activity of the ABI1 protein (Miyazono et al., 2009).

To date, the function of plant PP2Cs has been analyzed mainly by biochemical, molecular, and other biological methods due to lack of any specific PP2C inhibitors; if such inhibitors were available, they would be extremely useful tools in plant research, being complementary to other resources that can mimic the ABA response (Kim et al., 2011; Okamoto et al., 2013; Rodriguez and Lozano-Juste, 2015; Takeuchi et al., 2016; Cao et al., 2017; Vaidya et al., 2017; Vaidya et al., 2019). Virtual screening approaches for mammalian PP2C inhibitors have been undertaken, but with modest success. However, so far, no specific plant PP2C inhibitor has yet been identified.

In various systems ranging from mammalian to plant cells, a group of drugs including okadaic acid, cantharidin, calyculin A, fostriecin, and tautomycetin have been used as PP1, PP2A, and PP2B protein phosphatase inhibitors (Luan, 2003). In mammalian cells, a small number of compounds are classified as PP2C inhibitors. Cyclic phosphopeptide inhibits a protein phosphatase 2Cδ (PP2Cδ), while Evans blue and derivatives inhibit two isoforms of Ca^2+^/calmodulin-dependent protein phosphatase 2C. In addition, sanguinarine, an alkaloid, shows PP2Cα inhibitory activity and is reported to inhibit dual-specific PP2Cs including mitogen-activated protein phosphatase-1 (MKP-1) (Vogt et al., 2005; Rogers et al., 2006; Aburai et al., 2010). These compounds have not been tested in plants due to significant diversity between the respective plant and mammalian PP2C domain structures.

The recent definition of the ABI1 crystal structure and the conformational changes associated with ABA-PYL-ABI1 interactions should facilitate the development of efficient and specific plant PP2C inhibitors. Here we identify and characterize candidate inhibitors of group A PP2Cs in Arabidopsis, whose design is based on the mechanism of inhibition of ABI1 by ABA receptor PYL1. Using a two-step strategy that combines biochemical and *in silico* docking approaches we identified two compounds as candidate inhibitors of ABI1 protein phosphatase that have significant potential for manipulation of the ABA response.

## Materials and Methods

### Software

PyMol (DeLano, 2002) and Chimera (Goddard et al., 2018) were employed to visualize, analyze, and prepare figures of the molecular results obtained. The Schrödinger software package (Schrodinger suite; www.schrodinger.com) was used for all steps of the structure-based virtual screening including protein and ligand preparation, binding site detection, grid generation, molecular docking, and estimation of binding energy.

### Protein Structure Preparation

Three-dimensional structures of the group A protein phosphatase PP2C ABI1 in complex with the PYL1 receptor were retrieved from Protein Data Bank (PDB: 3KDJ and 3NMN) (Yin et al., 2009; Melcher et al., 2010). The PP2C protein structure was prepared for structure-based virtual screening and molecular docking by removing all water molecules, adding hydrogen atoms, and generating protonation states at pH 7. Manganese/magnesium (Mn^2+^ or Mg^2+^) ion ligands were included in the computation. The target protein was prepared by Protein Preparation Wizard implemented in the Schrodinger suite (Schrödinger Release 2019). The resulting structure was minimized using the molecular mechanics method of the Prime module of the Schrödinger Suite (Schrödinger Release 2019) with the OPLS2005 force field set to default convergence criteria (RMS gradient value equals 0.3).

### Chemical Compound Library Preparation

Approximately twenty-two million commercially available chemical compounds in the ZINC database (Irwin and Shoichet, 2005) were used for the initial docking studies. ZINC, which was retrieved in Sdf file format, contains millions of small ligands, grouped into subsets and classes depending on the mass, including Lead-like compounds, Fragment-like compounds, and Drug-like compounds. Ligand preparation was performed with the Ligprep module (Schrödinger Release 2019). Tautomeric states, ionization states, and also metal-binding states were generated using Epik (Schrödinger Release 2019).

### Binding Site and Grid Generation

The grid box was placed over the substrate-based inhibitor binding pocket shown in the crystallographic structure (PDB: 3KDJ and 3NMN) so as to cover the entire enzyme binding site and to allow ligands to move freely inside. The area of the binding site was determined and marked using SiteMap (Schrödinger Release 2019). For ABI1 from the 3KDJ crystal structure the grid box had the following parameters: box size dimensions of 27.88 × 27.88 × 27.88 (x, y, z); coordinates of x = −8.67909090909 Å, y = −2.46909090909 Å, z = 16.6127272727 Å at the center of the box. For ABI1 from the 3NMN crystal structure the grid box had the following parameters: box size dimensions of 30.51 × 30.51 × 30.51 (x, y, z); coordinates of x = −16.8238888889Å, y = 3.65111111111Å, z = 4.92444444444 Å at the center of the box. The grid boxes were generated using the Glide module (Schrödinger Release 2019).

### Molecular Docking

For docking studies, Glide software was used. The target protein was maintained rigid throughout the docking process, while the ligands were allowed to be flexible. Glide filters the molecules using HTVS (high-throughput virtual screening), SP (standard precision), and XP (extra precision) modes. OPLS2005 force field (Schrödinger Release 2019) parameters were applied while performing all steps of the docking calculations. All receptor-ligand structures obtained were scored and ranked according to docking score value. For each ligand at least 10 alternative poses were generated. During XP docking mode, the interactions value per residue was obtained.

### Protein-Ligand Complex Energy Minimization

Energy minimization for top ranked protein-ligand structures was performed using the Prime module (Schrödinger Release 2019). OPLS2005 force field atom typing parameters were added during this computation. Each minimization structure was used in further analysis.

### Binding Energy Estimation

Calculation of interaction energy was performed using Molecular Mechanics combined with Generalized Born and Surface Area continuum solvation (MM/GBSA) estimation procedure using the Prime module from the Schrödinger Suite (Schrödinger Site). For this computational method, a model of a single ligand complexed with ABI1 protein was used. During calculation all interaction residues were rigid and the entropic term was ignored. OPLS2005 force field (Schrödinger Release 2019) parameters were applied while performing this computation.

### Vector Construction, Protein Overexpression, Purification, and Quantitative Phosphatase Assays

Recombinant His- and GST-tagged ABI1 and ABI2 constructs were prepared using Gateway technology. cDNA for ABI1 was amplified using *Pfu* polymerase and cloned into the pENTR/SD/D-TOPO vector (Invitrogen) (Ludwikow et al., 2014). Next, the pENTR-ABI1 construct was recombined with the Gateway^®^ pDEST™15 or Gateway^®^ pDEST™17 vector to generate GST- or His-tagged ABI1, respectively. The resulting vector construct was transformed into *E. coli* BL21 (DE3) LysS and 0.5 mM isopropyl-β-D-1-thiogalactopyranoside was added to the bacterial culture, which had been grown to OD_600_ 0.8–1.2 at 37°C (~4 h), to induce expression of recombinant His-ABI1 protein. The bacterial pellet was suspended in PBS supplemented with an EDTA-free protease inhibitor cocktail together with phenylmethanesulfonyl fluoride and then sonicated. The supernatant was loaded on a Ni-NTA (Invitrogen) or glutathione-Sepharose column (GE Healthcare) according to the manufacturer’s protocol. After elution with imidazole or reduced glutathione respectively, the recombinant proteins were further separated using an Äkta™ pure M HPLC system, equipped with a Superdex™ 200 column for buffer exchange to the phosphate assay reaction buffer. Recombinant proteins were checked by SDS–PAGE and Coomassie Brilliant Blue staining. Purified proteins were subsequently used in the nonradioactive Serine/Threonine Phosphatase Assay System (Promega), according to the protocol described in (Ludwikow et al., 2014) and (Mitula et al., 2015). Phosphatase assay reactions were set up in 50 μl in a buffer of 20 mM HEPES pH 7.5, 20 mM MgCl_2_, 150 mM NaCl, 100 μM Ser/Thr phosphopeptide substrate, and 3 μg of PP2C. Kinetics analysis of enzyme-substrate was performed with 3 µg of ABI1 and increasing substrate concentration from 0 to 200 µM in two independent replicates. K_m_ value was calculated by nonlinear regression analysis. Candidate inhibitors were purchased from outside vendors.

### Cell-Free Degradation Assay

The cell-free degradation assay was performed as described in Marczak et al. (2020). In brief, 7-day-old WT Arabidopsis seedlings were treated with 100 µM MG132, 300 µM ZINC59151964, or 300 µM ZINC05273880. At least 500 µg total protein extract was incubated with 300 µg GST-ACS7. The GST-ACS7 protein level was monitored at the indicated intervals using anti-GST antibody (1:5,000, MoBiTec). An equal amount of solvents for each compound was used as mock-treatment controls. The intensity of the bands was quantified using ImageJ software.

### Germination Assay

For the germination assay, seeds of WT Coland *snrk2.2/snrk2.3* (GABI-KatC807G04/Salk_107315) mutant lines were surface sterilized and planted on half-strength, solid MS medium (with 1% sucrose) supplemented with or without 1 μM +/− ABA, ZINC59151964, and ZINC05273880 compound. The ABA was added to medium after sterilization. To avoid degradation of the compound, 150 μl was added on the surface of solid ½ MS medium and left for 1 h to soak in. After sowing, seeds were stratified for three days in the dark at 4°C, then transferred to a growth chamber and grown as described previously in Ludwików et al. (2009).

## Results

### Strategy for Identification of Novel Inhibitors

**Figure 1:**
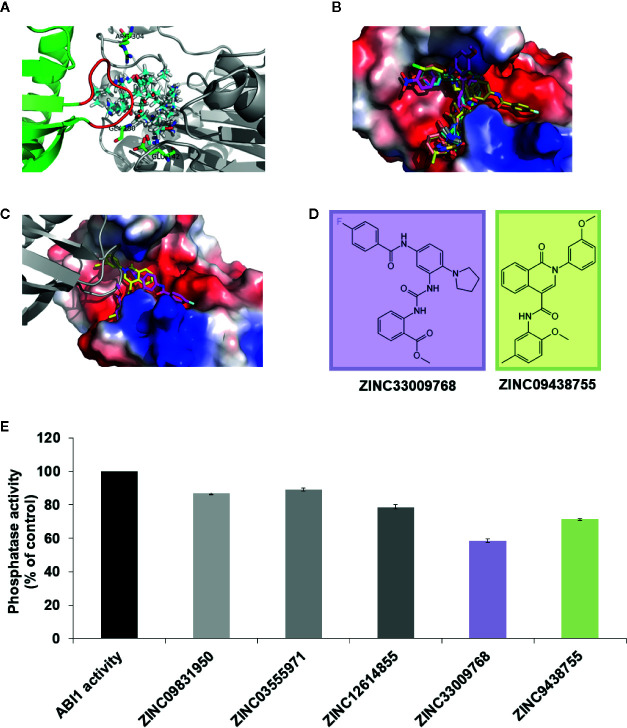
Initial docking study. **(A)** Protein target ABI1 from crystallographic structure PDB code 3KDJ (complex of PYL1-ABI1) showing proposed binding site (cyan-red-blue sticks) with residues crucial for the interaction labeled; **(B)** Fifteen chemical compounds from our docking studies were chosen for validation of ABI1 phosphatase activity; **(C)** In the ABI1 active site, the docking pose of ZINC33009768 is shown as a magenta backbone, and the docking pose of ZINC09438755 is shown as a yellow backbone. PYL1 is shown in gray (the gate-loop of PYL1 is visible adjacent to each chemical compound ZINC33009768 and ZINC09438755); **(D)** Chemical structure of ZINC09438755 and ZINC33009768 compounds generated in Maestro (Schrödinger Suite); **(E)** Effect of ZINC33009768 and ZINC09438755 on ABI1 protein phosphatase activity. Normalized results of phosphatase activity are shown in the presence of ZINC09438755 and ZINC33009768, respectively. The enzyme reactions were performed in a 50 µl final volume containing 3 µg of His–ABI1 with or without 100 μM ZINC33009768 or 100 μM ZINC09438755. The results presented are the means from three independent biological replicates.

To identify candidate group A PP2C inhibitors, we used a two-step strategy that combined molecular docking and biochemical assays. For *in silico* docking experiments, the crystal structure of ABI1 in complex with PYL1 (PDB: 3KDJ) (Yin et al., 2009) was used, due to the lack of an available crystal structure of full-length ABI1 alone. Based on the structural mechanism of inhibition of ABI1 by ABA receptor PYL1 (Yin et al., 2009), a putative binding site was proposed. In our first approach, we focused mainly on the structure of the ABI1-PYL-ABA ternary complex, which gives critical insights into ABI1-PYL binding. We screened for candidate inhibitors that bind *via* hydrogen bonds to the Gly180 and Glu142 residues, which are critical for ABI1 activity, are located at the active site, and are crucial for PYL1 binding to this phosphatase (**Figure 1A**). Any potential inhibitor should mimic the interaction between the PYL1 loop and ABI1. Candidate inhibitors would thus block the ABI1 active site and reduce substrate access, thereby slowing down the rate of its dephosphorylation reaction. Accordingly, a molecular docking site was constructed. For the first virtual screen, both a medium-size (around four million Lead-Like compounds from the ZINC database) (Irwin and Shoichet, 2005; Irwin et al., 2012) and a small library were used. The small library was generated for this analysis and consists of known phosphatase inhibitors (okadaic acid, tautomycin, microcystin, cantharidin, and sanguinarine chloride) (Swingle et al., 2007; Gotoh and Negishi, 2015) of protein phosphatases from different groups and their analogs. The 3D coordinates of the compiled compounds were downloaded from the ZINC12 database (Sterling and Irwin, 2015) with their protonation states, and atomic charges were assigned. Using this screening regime, we chose the 15 compounds with the highest binding scores from the medium-size database for experimental validation (**Figures 1B, C**). No compounds from the small library were selected.

**Figure 2:**
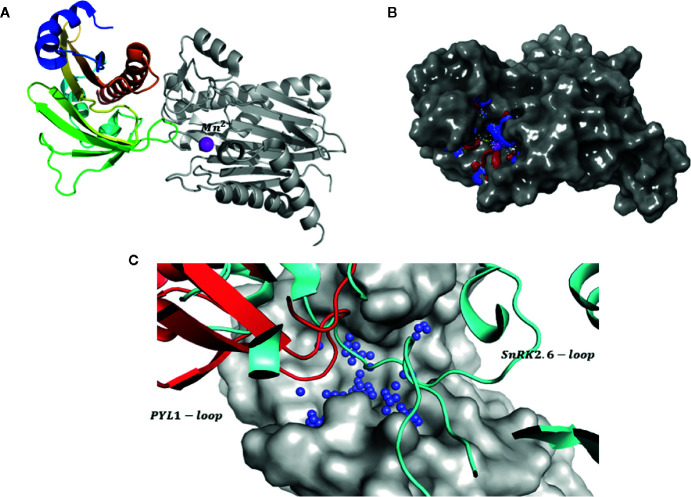
Strategy for identification of novel inhibitors. **(A)** Structure of PYL1-ABI1 complex (PDB code: 3KDJ). PYL1 is shown in color scale, while ABI1 PP2C is shown in grayscale. The manganese ion is shown as a pink sphere; **(B)** Target (gray surface) and a putative binding site (shown as blue red mesh): a metal ion is included at the binding site; **(C)** Comparison between proposed binding site (blue spheres) and interaction surface of PYL1 (shown as red cartoons) and SnRK2.6 (shown as light blue cartoons) and ABI1 (shown in gray surface representation).

To validate potential inhibitory activity, a protein phosphatase assay was performed on the compounds recovered from the first *in silico* screen. Only two molecules ZINC33009768 and ZINC09438755 were found to have significant inhibitory activity. However, the inhibition rate for these compounds was not satisfactory (**Figures 1D, E**). The known structure of an ABI1 homologue from PP2C group A to HAB1 with SnRK2.6 kinase substrate (PDB: 3UJG) (Soon et al., 2012) highlights the mechanism of dephosphorylation and therefore could be helpful in designing our docking studies. Taking this and the above preliminary experimental results into account prompted us to increase the docking region for potential inhibitors (**Figure 2C**).

Thus, a second *in silico* screen was carried out by extending the inhibitor binding site to include the interaction sites of both PYL and SnRK2.6 kinase, as well as part of the enzyme catalytic pocket that binds a metal ion (cofactor binding pocket) (**Figure 2A**). In our modeling strategy the structure of ABI1 PP2C (PDB: 3KDJ) (Yin et al., 2009) that binds manganese ion (Mn^2+^) was used and this ion was included in computations. Using this approach, we increased the possibilities for interaction between the protein and candidate inhibitors; we also added an extra scoring function to include the metal interaction term. We suggest that the metal ion in the catalytic pocket might be important for mediating protein-ligand binding, and therefore the predicted strength of binding between protein and candidate inhibitor should increase in the presence of this ion.

### Validation of Docking Hypothesis

**Figure 3:**
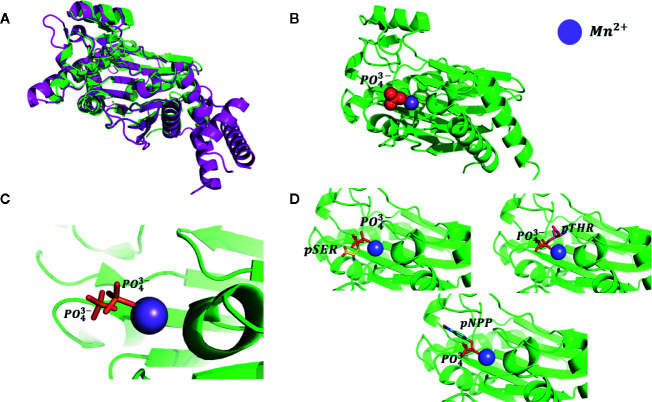
Testing of docking model. **(A)** Superposition of human PP2Ca structure (shown as magenta cartoons, PDB: 4RA2) and plant group A PP2C (shown as green cartoons, PDB: 3KDJ); **(B)** Monophosphate ion binding pose. Ions shown as spheres; the position of the monophosphate ion was taken from the superposition of those two phosphatase structures; **(C)** Redocking pose of the monophosphate ion using our proposed binding model and comparison with position of the monophosphate ion present in mammalian phosphatase PP2Ca; **(D)** Docking pose of pSER, pTHR, and pNPP. Docked molecules are shown as stick models.

To confirm our hypothesis, docking computations were performed using free phosphatase ion (P0_4_^3-^) and phosphoamino substrates like phosphoserine (pSER), phosphothreonine (THR), and para-nitrophenyl phosphate (pNPP) as ligands. These compounds were docked into the proposed binding site (**Figure 2B**). The calculated orientation of the phosphate group in the ABI1 protein matched very well that of the free phosphate ion in the crystal structure (PDB:4RA2) (**Figure 3A**) (Pan et al., 2015) of a mammalian PP2C with a co-crystallized phosphate ion (**Figures 3B–D**). Glide docking obtained similar bindings for all four molecules mentioned above with the lowest binding energy predictions (**Figure 3D**).

### High-Throughput Virtual Screening Procedure

**Figure 4:**
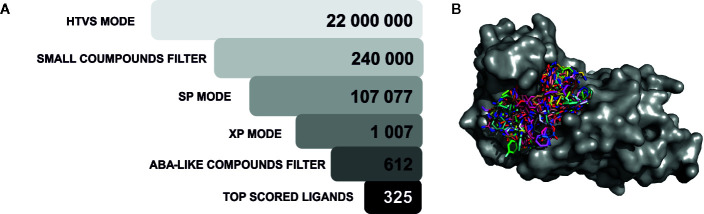
Virtual screening procedure. **(A)** The 325 top-ranked compounds resulting from the virtual screening procedure on a target ABI1 protein surface are shown. Selected molecules are shown as stick models on the protein surface; **(B)** The virtual screening procedure is shown as a set of filtering procedures, which gradually remove chemical compounds in each step. Initially, 22 million chemical compounds from the ZINC database were considered, and these were ultimately reduced to 325 candidate molecules. A three-step docking algorithm was used with different precision modes (HTVS, SP, XP). Small compounds were removed on the basis of number of atoms and ABA-like molecules were also filtered out computationally.

The generated docking site (see **Figure 2B**) was used in the second screening procedure (**Figure 4**). High-throughput virtual screening (HTVS) was carried out for ∼22 million compounds from the ZINC dataset (Irwin and Shoichet, 2005; Sterling and Irwin, 2015). Two hundred forty thousand compounds were top-ranked after HTVS of these, around 130,000 small compounds (molecules with less than 40 atoms) were filtered out. A standard precision (SP) docking approach was then applied to around 110,000 compounds (**Figure 4**). After the SP step, around 1,000 compounds were processed using a stricter docking approach, i.e. extra precision (XP). For the HVTS and SP screening stages, the 3KDJ structure of ABI1 (Yin et al., 2009) was used. During XP mode, two ABI1 target structures from different crystallographic experiments co-crystalized with Mg^2+^ (PDB: 3NMN) (Melcher et al., 2010) or Mn^2+^ (PDB: 3KDJ) (Yin et al., 2009) were used, mainly to allow side chain movement and subtle fluctuations in the geometry of the catalytic pocket (). The XP step produced 10 poses per compound, which were scored and ranked. The top-scoring ligands (612 molecules) from the XP mode docking experiments for both ABI1 targets, which had consensus docking scores less than −6.0 (the Glide score), were chosen and processed with an ABA-like filter. This procedure allows compounds with a chemical structure similar to that of ABA to be removed from the screen. Using this filtering strategy, the top-ranked candidates selected as ABI1 inhibitors should not bind to the ABA receptor. Thus, compounds with high scores for binding to the ABI1 protein, but also high for PYL1-receptor binding (Glide scores less than −4.0), were filtered out. This virtual screening strategy yielded 325 chemical compounds as potential ABI1 inhibitors.

### Chemical Compound Clustering and Induced-Fit Docking

Only 238 molecules from the selected set were commercially available. Using Canvas software, which is available in the Schrödinger package, our selected molecules were clustered to find similar compounds. Clustering was based on linear, pairwise, torsion, and triplet fingerprints. A leader-follower clustering algorithm was performed on the chemical compounds based on their calculated fingerprints. In each cluster set obtained, similar compounds were removed. Based on these results and visual inspection, 40 compounds were filtered out, leaving 198 molecules for induced-fit docking, which was implemented in Schrödinger. This procedure allows the docking pose of a ligand with flexible residues to be predicted in the protein binding site. The induced-fit protocol generated up to 80 docking poses for each ligand, and residues within 5 Å of the docking pose were refined. A grid box was defined based on the best binding pose of each compound in XP mode. Computation was performed using an OPLS2005 force field. All ligands were scored and ranked. In addition, we also performed interaction energy analysis with residues in the active site, mostly to quantify the interaction with crucial amino acids. The above computations allowed us to select 10 commercially available compounds for empirical validation (**Table 1**).

**Table 1:** Physicochemical and computational properties of experimentally validated compounds.

Compound ID	Docking score(kcal/mol)	Rank	Binding energy **(kcal/mol)	Inhibition ratio	Error rate	HBA	HBD	MW	PSA	RB
**ZINC26376363**	−9.061	18	−81.17	0.143	0.002	3	1	451.97	156.14	12
**ZINC59151964**	−8.017	75	−60.04	0.718	0.03	8	5	599.66	207.48	13
**ZINC08214766**	−8.725	32	−38.45	0.002	*	15	6	662.42	343.54	11
**ZINC29221575**	−8.755	24	−59.41	0.02	*	8	9	749.90	343.19	25
**ZINC13543740**	−6.948	325	−75.50	0.278	0.1	3	3	488.61	222.35	15
**ZINC71788455**	−7.433	151	−57.04	0.279	0.03	4	4	558.63	230.95	18
**ZINC02517180**	−7.689	111	−54.25	0.257	0.04	4	3	380.42	116.05	6
**ZINC71788395**	−8.706	33	−73.92	0.155	0.06	7	4	612.72	190.91	12
**ZINC03938642**	−6.967	257	−59.71	0.046	0.02	5	7	680.77	335.81	23
**ZINC05273880**	−7.741	105	−75.72	0.896	0.07	7	3	640.73	226.91	13
**ZINC13541100**	−8.603	38	−62.07	0.125	0.02	5	6	482.51	191.12	13
**ZINC04545840**	−8.739	50	−38.34	0.426	0.1	3	6	351.35	166.92	10

The Table shows some of the basic physicochemical properties of each molecule, such as molecular weight (MW), number of rotatable bonds (RB), number of hydrogen bond acceptors and donors (HBA, HBD), polar surface area (PSA). Theoretical data (docking score energy interaction value) and experimental ratio of inhibition are also listed. * very low value; ** estimation using MM-GBSA method.

### Validation of ABI1 Candidate Inhibitors

**Figure 5:**
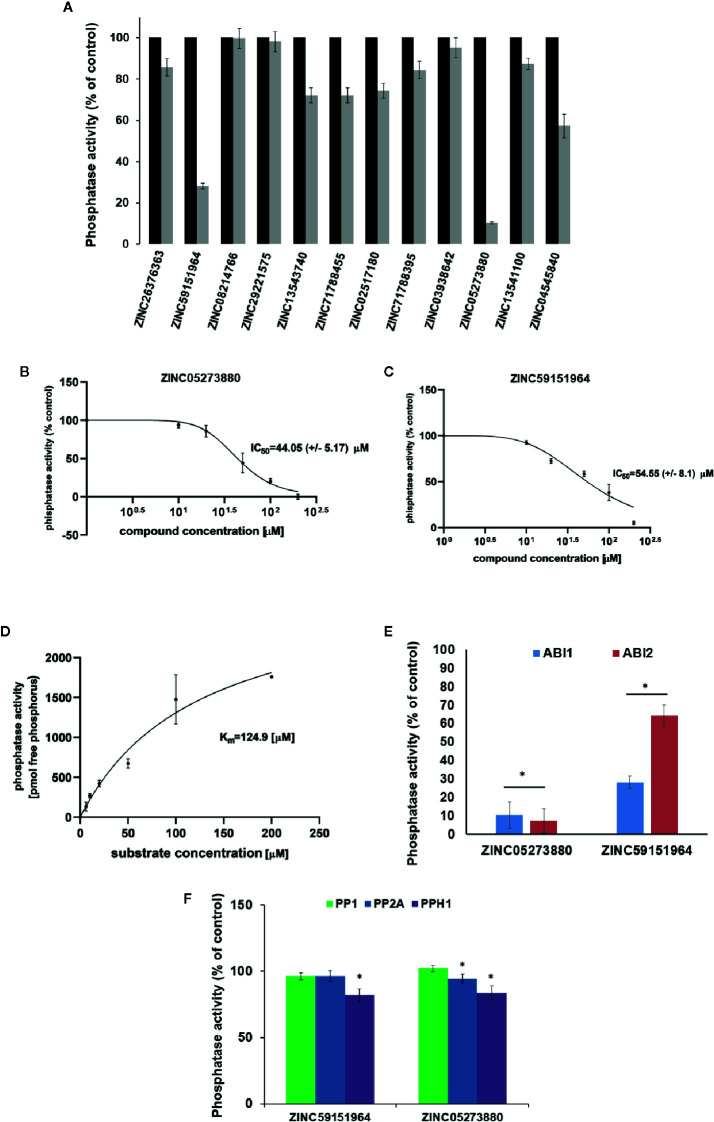
Effect of Candidate Inhibitors on ABI1 Protein Phosphatase Activity. **(A)** Normalized results of phosphatase activity in the presence of each potential PP2C inhibitor are shown. Phosphatase activity was obtained from three independent biological replicates. IC50 was determined for six different concentrations of ZINC05273880 **(B)** and ZINC59151964 **(C)**, respectively, in the range 0 to 200 µM. Results are an average value of two independent experiments (n = 12). IC50 was determined by setting the inhibition rate of phosphatase activity *versus* inhibitor concentration. Error bars represents standard deviation. **(D)** K_m_ value for ABI1 in a substrate range from 0 to 200 µM; **(E, F)** Effect of ZINC05273880 and ZINC59151964 on ABI1, ABI2 and non-clade A PP2Cs. Recombinant ABI1, ABI2, PP1, PP2A, and PPH1 proteins were assayed with (100 µM) and without (control) the indicated ZINC compounds. Values are expressed as a percentage of control and as the mean of two independent experiments (n = 6–12). The asterisks marks a significant difference by Student’s t-test (p < 0.001).

**Figure 6:**
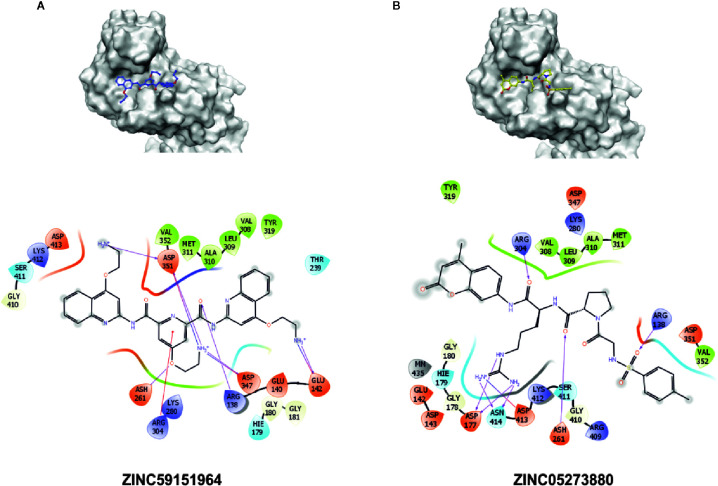
Interaction sites of best experimentally validated compounds. **(A)** ZINC05273880 interaction diagram and proposed binding pose with the crucial interactions between amino acid residues and the docked molecule highlighted; **(B)** ZINC59151964 interaction diagram and proposed binding pose with the crucial interactions between amino acid residues and the docked molecule highlighted. Amino acids within a 5 Å radius of the docking pose of the chemical compound are labeled.

**Figure 7:**
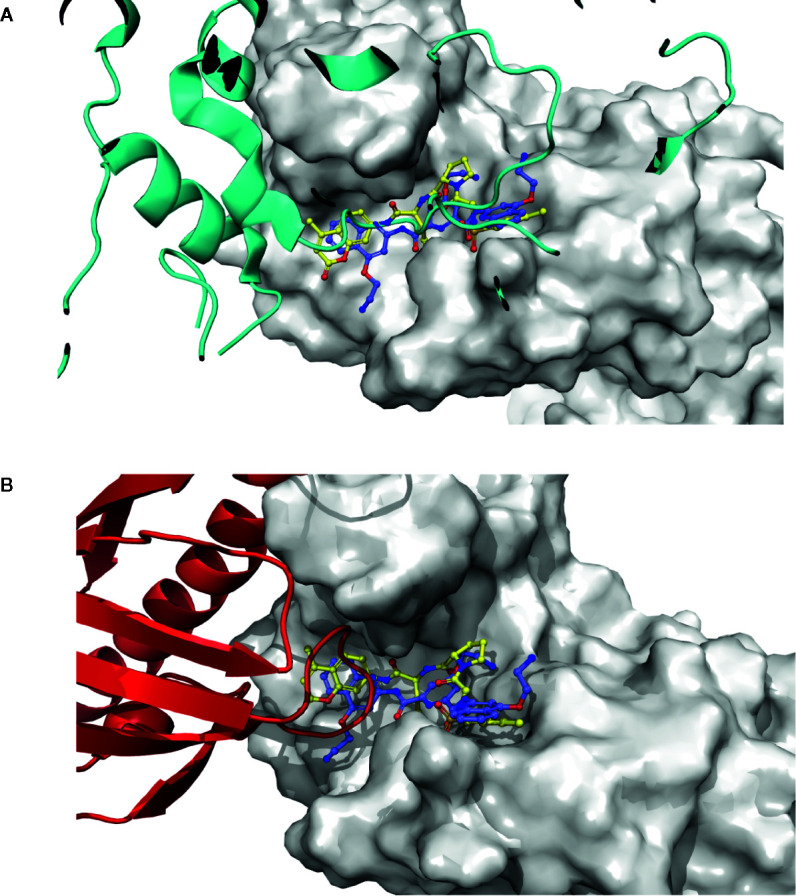
PYL1 and SnRK2.6 interaction loops, and docking pose structural correlation. **(A)** SnRK2.6 is shown as a blue cartoon, docked molecules are shown as sticks, and PP2C is shown as a surface. **(B)** The PYL1 receptor is shown as a red cartoon, docked molecules are shown as sticks, and PP2C is shown as a surface.

To test whether the selected 12 compounds inhibit ABI1 protein phosphatase activity *in vitro*, a phosphatase assay was performed with Ser/Thr phosphopeptide as an artificial substrate. Of the 12 compounds tested, two produced a marked decrease in ABI1 protein activity: at 100 µM, ZINC05273880 and ZINC59151964 resulted in 71 and 95% inhibition of ABI1, respectively. Other compounds inhibited ABI1 to a lesser extent, e.g. ZINC04545840 gives 42.6% inhibition while ZINC13543740, ZINC71788455, ZINC02517180 produced 25% inhibition (**Table 1**; **Figure 5A**). IC50 values for ZINC05273880 and ZINC59151964 were 44 ± 5 and 54 ± 8 µM, respectively (**Figures 5B, C**). K_m_ value for ABI1 was 124.9 µM (**Figure 5D**). A selectivity test against homologous protein phosphatase ABI2 showed that ZINC05273880 inhibits ABI1 and ABI2 at comparable levels, while ZINC59151964 is more efficient against ABI1 than ABI2 (**Figure 5E**). Experiments with non-clade A PP2Cs PP1, PP2A, and PPH1 (**Figure 5F**) showed that ZINC59151964 and ZINC05273880 inhibit PPH1 phosphatase activity to similar extents (up to 18%). ZINC05273880 also had some inhibitory effect (~6%) on PP2A activity. Computer docking predicted that both ZINC05273880 and ZINC59151964 interact with the active site of ABI1 and overlap the designed docking site (**Figures 6**, **7**).

**Figure 8:**
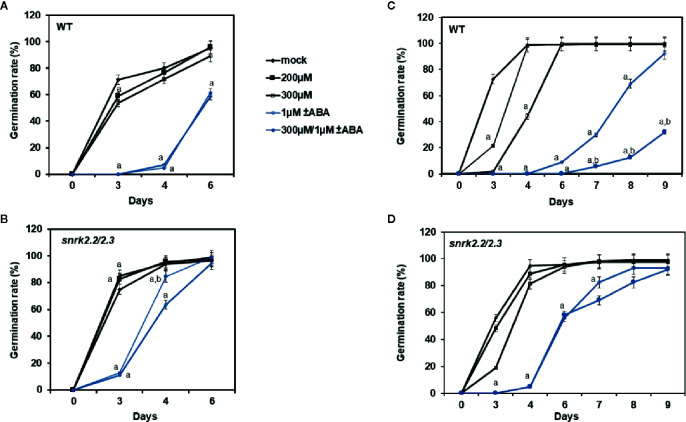
Effect of ZINC59151964, ZINC05273880 and ABA on seed germination. Seeds of WT **(A, C)**, and *snrk2.2/2.3* **(B, D)** mutant lines were grown on MS medium supplemented with ABA, the indicated concentration of ZINC compound or both. Right panel **(A, B)** presents germination results for ZINC59151964. Left panel **(C, D)** presents germination results for ZINC05273880. Seeds are considered germinated when green cotyledons have expanded. Values are the mean germination frequency from three to four separate plates (22–32 seeds per plate) for each genotype. Error bars indicate SD; “a” indicates a significant change (*P* < 0.05, Student’s *t*-test) compared with the mock control; “b” indicate a significant change (*P* < 0.05, Student’s *t*-test) between ABA and ABA/inhibitor treatment.

To test the usefulness of the candidate inhibitors *in vivo* we performed a germination assay. At a concentration of 200 µM over 3 days, ZINC59151964 and ZINC05273880 suppressed the germination rate of wild-type (WT) plants by 12.66% (p < 0.032) and 30% (p < 0.001), respectively (**Figures 8A, C**). A significant effect was also observed for ZINC05273880 at a concentration of 300 µM, which almost completely abolished germination of WT seeds (p < 0.0001). At the same time point (3 days), ABA treatment resulted in severe inhibition of WT seed growth. In contrast, 200 and 300 µM ZINC59151964 increased the germination rate by 8.5% (p < 0.030) and 10% (p < 0.037), respectively, in *snrk2.2/2.3* plants compared to WT mock treatment (**Figure 8B**). This effect was even more pronounced when 300 µM ZINC05273880 was applied: we observed a 20% increased germination rate for the *snrk2.2/2.3* mutant plants (p < 0.001) compared to WT plants at the 3-day time point. Furthermore, a combination of ABA and ZINC59151964 treatment significantly suppressed the germination rate of the double mutant (p < 0.0001) with a level of inhibition similar to that of ABA alone. A combination of ABA and ZINC05273880 treatment for the same time also abolished germination of WT and *snrk2.2/2.3* mutant seeds (**Figures 8C, D**). Interestingly, at the 4-day time point, we observed a 20% increase in germination rate of the ABA/ZINC59151964-treated double mutant (p < 0.017) compared with ABA alone. Simultaneous treatment with ABA and ZINC05273880 decreased germination rate of the double mutant after seven to eight days (p < 0.01) compared to ABA (**Figure 8D**) suggesting that, in contrast to ZINC59151964, ZINC05273880 increases sensitivity to ABA.

**Figure 9:**
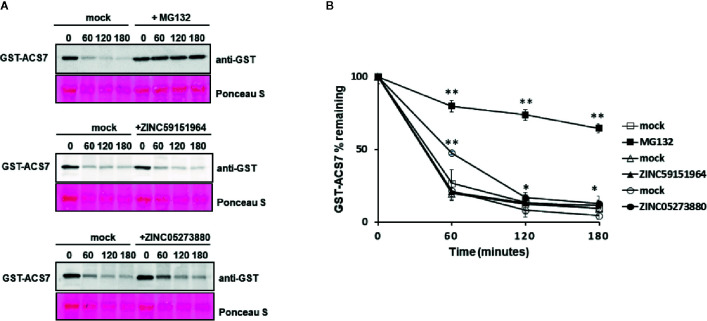
Cell-free degradation of recombinant GST-ACS7 protein. **(A)** Recombinant ACS7 protein was incubated with WT Col-0 protein extracts treated with or without MG132, ZINC95151964, and ZINC05273880 for the indicated times. The GST-ACS7 level was visualised by immunoblotting using anti-GST antibodies. Equal protein loading was shown by Ponceau S staining; **(B)** Half-life plot for cell-free degradation of ACS7 after MG132, ZINC95151964, and ZINC05273880 treatment. The GST-ACS7 bands were quantified using ImageJ software. Error bars indicate the SD (n = 4–6 replicates per time point) and the asterisks indicate a significant difference between mock and inhibitor treatment (based on Student’s t-test **p < 0.0001; *p < 0.03).

To further investigate the effect of ZINC59151964 and ZINC05273880 on PP2C function, we tested the stability of the ABI1/2 PP2C-interacting protein, ACC synthase 7 (ACS7) (Marczak et al., 2020), using a cell-free degradation assay (**Figure 9**). Recombinant GST-ACS7 was incubated with total protein extract prepared from WT Arabidopsis seedlings treated with 300 µM ZINC59151964, 300 µM ZINC05273880, 100 µM MG132 (a proteasome inhibitor), or an equivalent mock-treated control. As expected, degradation of GST-ACS7 was significantly delayed in plant extracts treated with MG132 (t_1/2_ > 180_ min_.). The stability of GST-ACS7 was also increased in extracts treated with ZINC05273880 (t_1/2_ ~60 min.), while no change in GST-ACS7 accumulation was observed after incubation with plant extracts treated with ZINC59151964.

## Discussion

ABI1 and ABI1-like PP2Cs are key elements in ABA signal transduction and regulate the plant adaptive response to environmental stress (Melcher et al., 2009; Ludwikow et al., 2014; Zhu, 2016). Using a two-step strategy combining biochemical and docking approaches, we identified two compounds (ZINC59151964 and ZINC05273880) as candidate inhibitors of ABI1, a group A PP2C in Arabidopsis. In nonradioactive phosphatase assays, these compounds gave around 75 and 95% inhibition of recombinant ABI1 indicating their potential use as *in vitro* ABI1 and ABI2 inhibitors. *In vivo* tests demonstrated that ZINC05273880 inhibits seed germination in WT and regulates stability of ACS7 protein in a cell-free degradation assay. In contrast, ZINC59151964 shows significant but limited inhibition of the germination rate of WT Col-0 and has no effect on ACS7 stability. A combined treatment with ABA and ZINC59151964, or treatment with ZINC59151964 alone, significantly improves the germination rate of *snrk2.2/2.3* at certain time points, which was an unexpected result. Overall, however, our analysis shows that ZINC05273880 is a promising candidate for future development as an inhibitor of ABI1 (and ABI2) for *in vitro* and *in vivo* use.

Among the strategies under consideration for crop improvement, manipulation of the ABA signaling pathway has become a significant target (Takeuchi et al., 2016). Much effort has been dedicated to the identification of synthetic compounds that mimic the effect of ABA. The most well-characterized are a tetrafluoro derivative of quinabactin (AMF4) pyrabactin, cyanabactin, and opabactin (Okamoto et al., 2013; Cao et al., 2017; Vaidya et al., 2017; Vaidya et al., 2019). Quinabactin is more efficient than pyrabactin in regulating the ABA response and is known to affect plant vegetative responses, especially adaptive responses that are essential for crop quality and quantity. Cyanabactin treatment significantly affects stomatal conductance and regulates ABA gene expression (Vaidya et al., 2017), while opabactin is currently the most effective tool for manipulating efficiency of water use (Vaidya et al., 2019; Lozano-Juste et al., 2020). Mandipropamid is another example of a compound that has the ability to activate an engineered PYR1 receptor and has significant potential for the modulation of ABA signaling in crops (Park et al., 2015; Rodriguez and Lozano-Juste, 2015). Another compound, Abz-E3M, is an inhibitor of abscisic acid 8′-hydroxylase and enhances the effect of ABA, leading to stomatal closure and enhanced ABA responses in both *Arabidopsis* and maize (Takeuchi et al., 2016). DFPM [5-(3,4-dichlorophenyl)furan-2-yl]-piperidine-1-ylmethanethione] downregulates ABA-dependent gene expression and also inhibits ABA-induced stomatal closure; it acts on a subset of ABA responses: for example, it does not affect the seed response (Kim et al., 2011). Last but not least, AA1, an antagonist of PYR/PYL-PP2C interactions, delays the ripening time of tomato in a dose-dependent manner, indicating its potential application in fruit storage (Ye et al., 2017). In contrast, the candidate inhibitors we have identified mimic the interaction between ABA receptors and ABI1 PP2C, and thereby block the active site and prevent access to substrates. We propose this strategy for manipulation of the ABA pathway as an alternative solution to those previously described (Kim et al., 2011; Okamoto et al., 2013; Rodriguez and Lozano-Juste, 2015; Takeuchi et al., 2016; Ye et al., 2017). *In vivo* inactivation of ABI1 PP2C activity is expected to enhance ABA responses in a similar manner to that observed in ABI1 knockout lines (Saez et al., 2006; Ludwików et al., 2009). Thus, in addition to their biotechnological potential, the two candidate inhibitors can be further optimized to make them more useful to dissect the chemical biology of PP2Cs.

Although they provide important insights into the mechanism of PP2C function, use of the available plant PP2C crystal structures as a docking model is not straightforward. Thus, the metal ion embedded in the PP2C active site directly coordinates three negatively charged residues as well as water molecules. This feature means that there are several possible models for docking, as any of the water molecules or the metal ion could be displaced upon inhibitor binding. Additionally, the PP2C crystal structure was determined with PYL1 receptor bound at the active site; it is not known whether the protein conformation changes substantially upon substrate or inhibitor binding. Due to differences between free and bound forms of a particular protein, structures without substrate or inhibitor bound are often less successful for docking studies (Pagadala et al., 2017). Nonetheless, the available crystallographic structures of ABI1 provided valuable information for molecular modeling.

The docking results for the diverse set of chemical compounds in the ZINC dataset allowed us to identify possible ABI1 inhibitors. The *in silico* stage of our study yielded nearly 200 molecules with potential affinity for the ABI1 protein. These compounds have high docking scores and binding energies (**Table 1**). *In vitro* phosphatase assay experiments validated two compounds, ZINC59151964 (4-(2-aminoethoxy)-N2,N6-bis[4-(2-aminoethoxy)-2-quinolinyl]-2,6-pyridinedicarboxamide) and ZINC05273880 (N-*p*-tosyl-Gly-Pro-Arg-7-amido-4-methylcoumarin), showing them to have significant inhibition ratios against ABI1. Compound ZINC59151964 is a symmetric molecule with two 2-aminoethoxy-2-quinolinyl organic moieties bridged by 2,6-pyridinedicarboxamide with an aminoethoxy tail in the four position. The quinolinyl groups bind in two opposite cavities inside the catalytic pocket. The first cavity consists of H179, G180, G181, V308, L309, and A310, while the second region is a shallow surface cavity comprising K412, S411, G410, and D351 (**Figure 6B**). The pyridinedicarboxamide core of this compound binds in the middle of the pocket, close to the metal binding active site. The oxygens from the carboxyl groups form hydrogen bonds with amino group hydrogens (R138), while the pyridine core interacts with R304 *via* a pi-cation interaction. In addition, a nitrogen heteroatom with a free electron pair forms a hydrogen bond with the amino group from R138. The three aminoethoxy tails, which are highly flexible, are also stabilized by electrostatic interaction between the positively charged NH_3_^+^ group and three negatively charged residues (D347, D351 and E142) (**Figure 6A**). D347 is involved in metal ion interaction in the active site. Compound ZINC05273880 is an oligopeptide containing three amino acids (GPR) connected by peptide bonds to tosyl and methylcoumarin groups. The arginine tail binds deeply inside the ABI1 active site and forms an electrostatic interaction with negatively charged E177 and E413, which coordinate the metal ion. The tosyl and methylcoumarin groups bind to the ABI1 protein in a similar fashion to the quinolinyl groups of compound ZINC59151964 except that a hydrogen bond is formed between the oxygen from a sulfonyl group and a hydrogen from R138, and a hydrogen bond is formed between a hydrogen from R304 and the oxygen from an amide group, which links the methylcoumarin group with the arginine tail of the compound (**Figures 6A, B**).

In summary, using virtual screening in combination with an *in vitro* phosphatase assay, we have discovered two molecules that inhibit ABI1 dephosphorylation activity. The compounds identified have different organic scaffolds and inhibit ABI1 at high micromolar concentrations. The physical properties of these compounds (**Table 1**) are drug-like, in terms of the rule of five (ROF) (omitting molecular mass) or the combination of rotatable bonds and polar surface area. Most importantly, some properties of the structures of these compounds, i.e. the pyridinedicarboxamide core, suggest possibilities for the development of related, but more potent, PP2C inhibitors.

## Data Availability Statement

The datasets generated for this study are available on request to the corresponding author.

## Author Contributions

AL and MJ conceived the project, designed the research, and wrote the paper. MJ, MM, and AC performed the experiments. AL, MJ, and AC analysed the data.

## Funding

This work was supported by the National Science Centre (NCN) of Poland grant no. 2016/22/E/NZ3/00345. No conflict of interest is declared.

## Conflict of Interest

The authors declare that the research was conducted in the absence of any commercial or financial relationships that could be construed as a potential conflict of interest.

## Abbreviations

ABA, abscisic acid; ABI1, abscisic acid insensitive1; MM/GBSA, molecular mechanics/generalized Born surface area; PP2C, protein phosphatase 2C; PYL1, pyrabactin resistance-like1 abscisic acid receptor; SnRK2, SNF1-related protein kinase 2; SP, standard precision docking; XP, extra precision docking.

## References

[B1] Aburai N. Yoshida M. Ohnishi M. Kimura K. (2010). Sanguinarine as a potent and specific inhibitor of protein phosphatase 2C in vitro and induces apoptosis via phosphorylation of p38 in HL60 cells. Biosci. Biotechnol. Biochem. 10.1271/bbb.90735 20208361

[B2] Bhaskara G. B. Nguyen T. T. Verslues P. E. (2012). Unique drought resistance functions of the highly ABA-induced clade a protein phosphatase 2Cs. Plant Physiol. 160, 379–395. 10.1104/pp.112.202408 22829320 PMC3440212

[B3] Cao M. J. Zhang Y. L. Liu X. Huang H. Zhou X. E. Wang W. L. (2017). Combining chemical and genetic approaches to increase drought resistance in plants. Nat. Commun. 8, 1183. 10.1038/s41467-017-01239-3 29084945 PMC5662759

[B4] Cutler S. R. Rodriguez P. L. Finkelstein R. R. Abrams S. R. (2010). Abscisic Acid: Emergence of a Core Signaling Network. Annu. Rev. Plant Biol. 61, 651–679. 10.1146/annurev-arplant-042809-112122 20192755

[B5] DeLano W. L. (2002). The PyMOL Molecular Graphics System (San Carlos, CA: DeLanoScientific). Available at: www.pymol.org.

[B6] Friso G. Van Wijk K. J. (2015). Posttranslational protein modifications in plant metabolism. Plant Physiol. 169, 1469–1487. 10.1104/pp.15.01378 26338952 PMC4634103

[B7] Fuchs S. Grill E. Meskiene I. Schweighofer A. (2013). Type 2C protein phosphatases in plants. FEBS J. 280, 681–693. 10.1111/j.1742-4658.2012.08670.x 22726910

[B8] Gerotto C. Trotta A. Bajwa A. A. Mancini I. Morosinotto T. Aro E. M. (2019). Thylakoid protein phosphorylation dynamics in a moss mutant lacking SERINE/THREONINE PROTEIN KINASE STN8. Plant Physiol. 180, 1582–1597. 10.1104/pp.19.00117 31061101 PMC6752907

[B9] Goddard T. D. Huang C. C. Meng E. C. Pettersen E. F. Couch G. S. Morris J. H. (2018). UCSF ChimeraX: Meeting modern challenges in visualization and analysis. Protein Sci. 27, 14–25. 10.1002/pro.3235 28710774 PMC5734306

[B10] Gotoh S. Negishi M. (2015). Statin-activated nuclear receptor PXR promotes SGK2 dephosphorylation by scaffolding PP2C to induce hepatic gluconeogenesis. Sci. Rep. 5, 14076. 10.1038/srep14076 26392083 PMC4585725

[B11] Himmelbach A. Hoffmann T. Leube M. Höhener B. Grill E. (2002). Homeodomain protein ATHB6 is a target of the protein phosphatase ABI1 and regulates hormone responses in Arabidopsis. EMBO J. 21, 3029–3038. 10.1093/emboj/cdf316 12065416 PMC126069

[B12] Irwin J. J. Shoichet B. K. (2005). ZINC - A free database of commercially available compounds for virtual screening. J. Chem. Inf. Model. 45, 177–182. 10.1021/ci049714+ 15667143 PMC1360656

[B13] Irwin J. J. Sterling T. Mysinger M. M. Bolstad E. S. Coleman R. G. (2012). ZINC: A free tool to discover chemistry for biology. J. Chem. Inf. Model. 52, 1757–1768. 10.1021/ci3001277 22587354 PMC3402020

[B14] Kim T. H. Hauser F. Ha T. Xue S. Böhmer M. Nishimura N. (2011). Chemical genetics reveals negative regulation of abscisic acid signaling by a plant immune response pathway. Curr. Biol. 21, 990–997. 10.1016/j.cub.2011.04.045 21620700 PMC3109272

[B15] Lozano-Juste J. García-Maquilón I. Ruiz-Partida R. Rodriguez P. L. (2020). Drug Discovery for Thirsty Crops. Trends Plant Sci. 25, 844–846. 10.1016/j.tplants.2020.07.001 32690361

[B16] Luan S. (2003). Protein phosphatases in plants. Annu. Rev. Plant Biol. 54, 63–92. 10.1146/annurev.arplant.54.031902.134743 14502985

[B17] Ludwików A. Kierzek D. Gallois P. Zeef L. Sadowski J. (2009). Gene expression profiling of ozone-treated Arabidopsis abi1td insertional mutant: Protein phosphatase 2C ABI1 modulates biosynthesis ratio of ABA and ethylene. Planta 230, 1003–1017. 10.1007/s00425-009-1001-8 19705149

[B18] Ludwikow A. Ciesla A. Kasprowicz-Maluski A. Mituła F. Tajdel M. Gałganski L. (2014). Arabidopsis Protein Phosphatase 2C ABI1 Interacts with Type i ACC Synthases and Is Involved in the Regulation of Ozone-Induced Ethylene Biosynthesis. Mol. Plant 7, 960–976. 10.1093/mp/ssu025 24637173

[B19] Marczak M. Cieśla A. Janicki M. Kasprowicz-Maluśki A. Kubiak P. Ludwików A. (2020). Protein Phosphatases Type 2C Group A Interact with and Regulate the Stability of ACC Synthase 7 in Arabidopsis. Cells 9, 978. 10.3390/cells9040978 PMC7227406 32326656

[B20] Melcher K. Ng L. M. Zhou X. E. Soon F. F. Xu Y. Suino-Powell K. M. (2009). A gate-latch-lock mechanism for hormone signalling by abscisic acid receptors. Nature 462, 602–608. 10.1038/nature08613 19898420 PMC2810868

[B21] Melcher K. Xu Y. Ng L. M. Zhou X. E. Soon F. F. Chinnusamy V. (2010). Identification and mechanism of ABA receptor antagonism. Nat. Struct. Mol. Biol. 17, 1102–1108. 10.1038/nsmb.1887 20729862 PMC2933329

[B22] Mitula F. Tajdel M. Cies̈la A. Kasprowicz-Malus̈ki A. Kulik A. Babula-Skowrońska D. (2015). Arabidopsis ABA-Activated Kinase MAPKKK18 is Regulated by Protein Phosphatase 2C ABI1 and the Ubiquitin-Proteasome Pathway. Plant Cell Physiol. 56 2351–2367. 10.1093/pcp/pcv146 26443375 PMC4675898

[B23] Miyakawa T. Fujita Y. Yamaguchi-Shinozaki K. Tanokura M. (2013). Structure and function of abscisic acid receptors. Trends Plant Sci. 18, 259–266. 10.1016/j.tplants.2012.11.002 23265948

[B24] Miyazono K. II Miyakawa T. Sawano Y. Kubota K. Kang H. J. Asano A. (2009). Structural basis of abscisic acid signalling. Nature 462, 609–614. 10.1038/nature08583 19855379

[B25] Nishimura N. Hitomi K. Arvai A. S. Rambo R. P. Hitomi C. Cutler S. R. (2009). Structural mechanism of abscisic acid binding and signaling by dimeric PYR1. Science 326, 1373–1379. 10.1126/science.1181829 19933100 PMC2835493

[B26] Nishimura N. Sarkeshik A. Nito K. Park S. Y. Wang A. Carvalho P. C. (2010). PYR/PYL/RCAR family members are major in-vivo ABI1 protein phosphatase 2C-interacting proteins in Arabidopsis. Plant J. 61, 290–299. 10.1111/j.1365-313X.2009.04054.x 19874541 PMC2807913

[B27] Okamoto M. Peterson F. C. Defries A. Park S. Y. Endo A. Nambara E. (2013). Activation of dimeric ABA receptors elicits guard cell closure, ABA-regulated gene expression, and drought tolerance. Proc. Natl. Acad. Sci. U. S. A. 110, 12132–12137. 10.1073/pnas.1305919110 23818638 PMC3718107

[B28] Pagadala N. S. Syed K. Tuszynski J. (2017). Software for molecular docking: a review. Biophys. Rev. 9, 91–102. 10.1007/s12551-016-0247-1 28510083 PMC5425816

[B29] Pan C. Tang J. Y. Xu Y. F. Xiao P. Liu H. Wang H. A. (2015). The catalytic role of the M2 metal ion in PP2Cα. Sci. Rep. 5, 8560. 10.1038/srep08560 25708299 PMC5390078

[B30] Park S.-Y. Peterson F. C. Mosquna A. Yao J. Volkman B. F. Cutler S. R. (2015). Agrochemical control of plant water use using engineered abscisic acid receptors. Nature 520, 545–548. 10.1038/nature14123 25652827

[B31] Rodriguez P. L. Lozano-Juste J. (2015). Unnatural agrochemical ligands for engineered abscisic acid receptors. Trends Plant Sci. 20, 330–332. 10.1016/j.tplants.2015.04.001 25891067

[B32] Rogers J. P. Beuscher IV A. E. Flajolet M. McAvoy T. Nairn A. C. Olson A. J. (2006). Discovery of protein phosphatase 2C inhibitors by virtual screening. J. Med. Chem. 49, 1658–1667. 10.1021/jm051033y 16509582 PMC2538531

[B33] Saez A. Robert N. Maktabi M. H. Schroeder J. II Serrano R. Rodriguez P. L. (2006). Enhancement of abscisic acid sensitivity and reduction of water consumption in Arabidopsis by combined inactivation of the protein phosphatases type 2C ABI1 and HAB1. Plant Physiol. 141, 1389–1399. 10.1104/pp.106.081018 16798945 PMC1533955

[B34] Saez A. Rodrigues A. Santiago J. Rubio S. Rodriguez P. L. (2008). HAB1-SWI3B interaction reveals a link between abscisic acid signaling and putative SWI/SNF chromatin-remodeling complexes in Arabidopsis. Plant Cell 20, 2972–2988. 10.1105/tpc.107.056705 19033529 PMC2613670

[B35] Schweighofer A. Hirt H. Meskiene I. (2004). Plant PP2C phosphatases: Emerging functions in stress signaling. Trends Plant Sci. 9, 236–243. 10.1016/j.tplants.2004.03.007 15130549

[B36] Soon F. F. Ng L. M. Zhou X. E. West G. M. Kovach A. Tan M. H. E. (2012). Molecular mimicry regulates ABA signaling by SnRK2 kinases and PP2C phosphatases. Science 335, 85–88. 10.1126/science.1215106 22116026 PMC3584687

[B37] Sterling T. Irwin J. J. (2015). ZINC 15 - Ligand Discovery for Everyone. J. Chem. Inf. Model. 55, 2324–2337. 10.1021/acs.jcim.5b00559 26479676 PMC4658288

[B38] Swingle M. Ni L. Honkanen R. E. (2007). Small-molecule inhibitors of ser/thr protein phosphatases: Specificity, use and common forms of abuse. Methods Mol. Biol. 365, 23–38. 10.1385/1-59745-267-X:23 17200551 PMC2709456

[B39] Takeuchi J. Okamoto M. Mega R. Kanno Y. Ohnishi T. Seo M. (2016). Abscinazole-E3M, a practical inhibitor of abscisic acid 8′-hydroxylase for improving drought tolerance. Sci. Rep. 6, 37060. 10.1038/srep37060 27841331 PMC5107945

[B40] Umezawa T. Nakashima K. Miyakawa T. Kuromori T. Tanokura M. Shinozaki K. (2010). Molecular basis of the core regulatory network in ABA responses: Sensing, signaling and transport. Plant Cell Physiol. 51, 1821–1839. 10.1093/pcp/pcq156 20980270 PMC2978318

[B41] Vaidya A. Peterson F. Yarmolinsky D. Merilo E. Verstraeten I. Park S.-Y. (2017). A rationally designed agonist defines subfamily IIIA ABA receptors as critical targets for manipulating transpiration. ACS Chem. Biol. 12, 2842–2848. 10.1021/acschembio.7b00650 28949512

[B42] Vaidya A. Helander J. Peterson F. Elzinga D. Dejonghe W. Kaundal A. (2019). Dynamic control of plant water use using designed ABA receptor agonists. Science 366, eaaw8848. 10.1126/science.aaw8848 31649167

[B43] Vogt A. Tamewitz A. Skoko J. Sikorski R. P. Giuliano K. A. Lazo J. S. (2005). The benzo[c]phenanthridine alkaloid, sanguinarine, is a selective, cell-active inhibitor of mitogen-activated protein kinase phosphatase-1. J. Biol. Chem. 280, 19078–19086. 10.1074/jbc.M501467200 15753082

[B44] Ye Y. J. Zhou L. J. Liu X. Liu H. Li D. Q. Cao M. J. (2017). A novel chemical inhibitor of ABA signaling targets all ABA receptors. Plant Physiol. 173, 2356–2369. 10.1104/pp.16.01862 28193765 PMC5373061

[B45] Yin P. Fan H. Hao Q. Yuan X. Wu D. Pang Y. (2009). Structural insights into the mechanism of abscisic acid signaling by PYL proteins. Nat. Struct. Mol. Biol. 16, 1230–1236. 10.1038/nsmb.1730 19893533

[B46] Yoshida R. Umezawa T. Mizoguchi T. Takahashi S. Takahashi F. Shinozaki K. (2006). The regulatory domain of SRK2E/OST1/SnRK2.6 interacts with ABI1 and integrates abscisic acid (ABA) and osmotic stress signals controlling stomatal closure in Arabidopsis. J. Biol. Chem. 281, 5310–5318. 10.1074/jbc.M509820200 16365038

[B47] Zhu J. K. (2016). Abiotic Stress Signaling and Responses in Plants. Cell 167, 313–324. 10.1016/j.cell.2016.08.029 27716505 PMC5104190

